# Annular one-dimensional photonic crystals for salinity sensing

**DOI:** 10.1038/s41598-023-47205-6

**Published:** 2023-11-23

**Authors:** Hassan Sayed, Mohamed A. Swillam, Arafa H. Aly

**Affiliations:** 1 TH-PPM Group, Physics Department, Faculty of Sciences, Beni-Suef University, Beni Suef, 62514 Egypt; 2 Department of Physics, The American University in Cairo, Cairo, Egypt

**Keywords:** Energy science and technology, Materials science, Optics and photonics, Physics

## Abstract

The use of annular one-dimensional (1D) photonic crystals (PCs) for salinity sensing is studied in this research. Annular 1D-PCs provide small and integrated structures that facilitate the creation of portable and miniaturized sensor equipment appropriate for field use. In order to generate annular 1D-PCs, the research explores the finite element method (FEM) simulation technique utilizing the COMSOL Multiphysics approach, highlighting the significance of exact control over layer thickness and uniformity. Furthermore, we construct a 1D annular PCs structure in the form ${(AB)}^{N}$, where A is silicon ($Si$) and B is silicon dioxide (${SiO}_{2}$) of 40 nm and 70 nm, respectively, with a number of periods equal to 9. By incorporating a central defect layer of saline water (220 nm thickness), the sensor achieves optimum performance at normal incidence with a sensitivity (S) of $782 (nm/RIU)$, a quality factor (Q) of 10.22, and a figure of merit (FOM) of $12.6 {RIU}^{-1}$. The design that is suggested has several advantages over past work on planners and annular 1D-PCs, including ease of implementation, performance at normal incidence, and high sensitivity.

## Introduction

In many areas, such as environmental monitoring, agriculture, and desalination processes, salinity is an important characteristic^1,2^. Thus, the creation of effective and trustworthy salinity sensors is crucial^3^. One-dimensional photonic crystals (1D PCs) have attracted a lot of attention recently as potential platforms for optical sensing applications^4–6^. Additionally, two-dimensional photonic crystals (2D PCs) have a broadband for salinity detection^7,8^, however, researchers and scientists prefer to employ one-dimensional photonic crystals (1D PCs) due to their benefits of being simple to make and having a number of uses. Hence, these structures from 1D PCs, comprising alternating layers of materials with high and low refractive indices, exhibit unique optical properties such as photonic bandgaps (PBG) that can be tailored to specific wavelengths of interest. One configuration of 1D PCs that has shown promise for salinity sensing is the annular geometry, where the layers are arranged in concentric rings. Numerous benefits of the circular design include improved sensitivity, compactness, and ease of production^9^. It is possible to optically detect changes in the local refractive index brought on by salinity variations by inserting ion-selective membranes or hydrogels into the annular 1D PC structure^10^. This offers a non-intrusive and label-free method for salinity sensing.

Annular 1D PCs offer unique advantages for salinity sensing applications^11–13^. The annular design, consisting of concentric rings of materials with a high and low refractive index, enables improved sensitivity and compactness compared to traditional planar configurations^14^. Due to the improved interaction between the sensing components and the incident light made possible by this design, the local refractive index changes brought on by salinity variations are more detectable^15^. An adaptable and scalable manufacturing process can be used to fabricate annular 1D PCs using methods including thin-film deposition, lithography, and etching^16^. Numerous elements can affect the performance of annular 1D PC salinity sensors, including the choice of material^17^, layer thicknesses, and the presence of ion-selective membranes or hydrogels. These factors can be optimized to achieve high sensitivity, selectivity, and stability^18,19^. Additionally, the compact and integrated nature of annular 1D PCs allows for the development of miniaturized and portable sensing devices^20,21^, suitable for field applications and real-time monitoring.

In annular 1D PC salinity sensors, the shift in the photonic bandgap is typically measured through optical characterization techniques^22–24^. These techniques rely on monitoring changes in the transmission or reflection spectra of the annular 1D PC structure. The reflected or transmitted light is then collected and analyzed using a spectrometer or a tunable filter^25^. The generated spectrum gives details about the sensor's photonic band gap. The local refractive index of the surrounding medium alters as the saline concentration does^26^, which results in a shift in the position or size of the photonic band gap. The reflected or transmitted light spectrum varies in accordance with this shift. The shift in the photonic band gap can be identified by contrasting the resulting spectrum with a reference spectrum captured at a known salinity condition. Overall, the shift in the photonic band gap of annular 1D PC salinity sensors may be precisely quantified by observing the variations in the reflected or transmitted light spectrum, offering a dependable and non-invasive way for salinity detection^27^. The potential applications of annular 1D PC salinity sensors are broad. They can be utilized in environmental monitoring to assess water quality in rivers, lakes, and coastal areas^28–30^. In agriculture, these sensors can aid irrigation management by monitoring soil salinity levels^31^, ensuring optimal crop growth and water usage. Furthermore, in desalination processes, annular 1D PC sensors can assist in monitoring the efficiency and effectiveness of desalination techniques, contributing to the development of sustainable water treatment solutions^32,33^.

The design and optimization of 1D-annular PCs as salinity sensors based on the location of the generated photonic band gap is the reason for this paper's originality. Here, we couple the location of the photonic band gap with the optical characteristics of the saline solution, as well as the thickness and location of the defect layer from the saline solution or other fluids, as it might be utilized as a biosensor. The numerous 1D annular PC structures may thus pinpoint and distinguish the various defect modes in the PBG regions with great accuracy. We will then evaluate the sensor performance by computing a number of parameters, such as the sensitivity (S), the figure of merits (FOM), and the quality factor (Q). In the manuscript's second section, we then demonstrate our design modeling. Finally, we present the findings and go over our salinity sensor.

## Theoretical analysis and modeling

**Figure 1:**
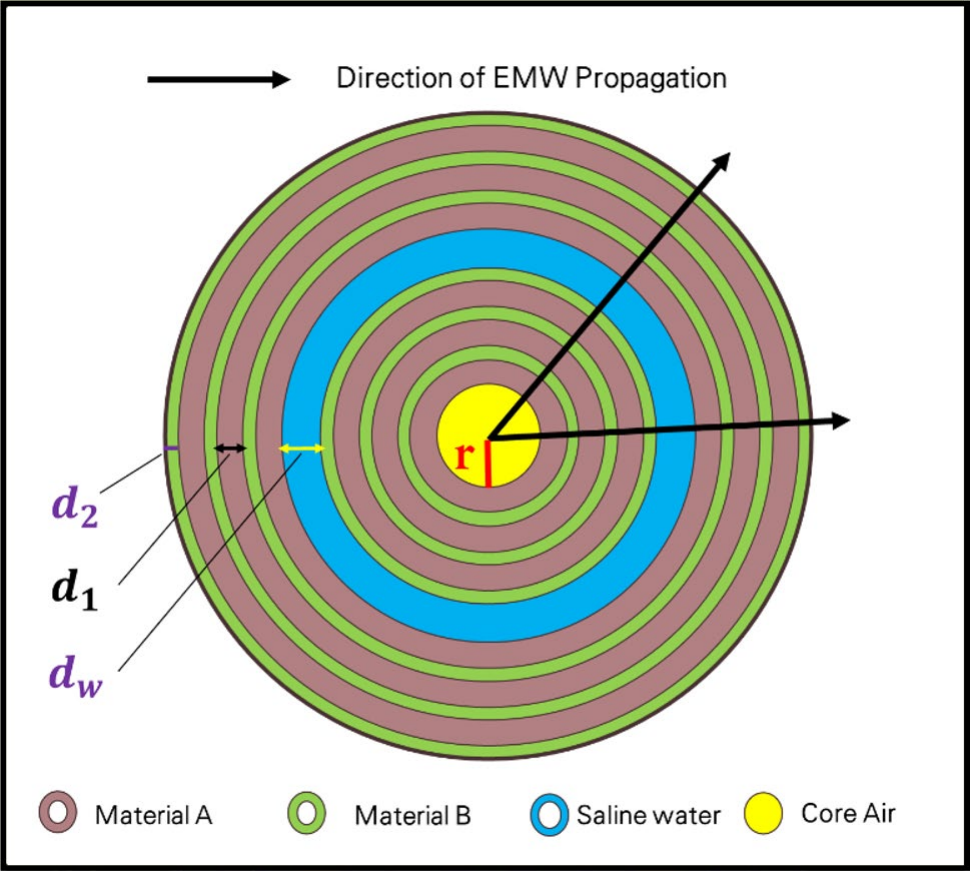
Schematic structure for a one-dimensional annular photonic crystal structure.

This section examines the theoretical modeling of our structure. As previously stated, our structure is simulated by COMSOL Multiphysics, which depends on the finite element method [FEM]^34^. As shown in Fig. 1, our design is an annular photonic crystal structure, which is considered a central cylinder coated with a periodic structure from two different materials; therefore, this structure is considered a one-dimensional photonic crystal as the periodic modulation is in one dimension, as shown.

Introducing the light source and detecting the spectrum in practice for a 1D annular PC structure, as shown in Fig. 1, which refers to the direction of the light propagation, could be achieved through several steps. Firstly, we select a suitable light source to illuminate the 1D annular PC structure. The choice of light source depends on the specific wavelength range of interest and the optical properties of the PC structure. Common light sources include lasers, light-emitting diodes (LEDs), or broadband sources such as white light sources or supercontinuum sources. In the second step, the light from the source needs to be coupled into the 1D annular PC structure. This can be achieved using optical fibers, lenses, or other coupling techniques. The specific coupling method may vary depending on the experimental setup and the type of light source used. Thirdly, this step involves measuring the intensity of the transmitted light as a function of wavelength. The transmission can be measured directly by placing a detector on the output side of the PC structure. Alternatively, you can collect the transmitted light and direct it to a spectrometer or an optical spectrum analyzer to obtain a detailed spectral profile. Finally, we analyze the obtained transmitted spectrum. This may involve identifying the resonant modes or features associated with the annular PC structure. By analyzing the spectral response, we can determine the wavelength shifts or changes induced by different factors, such as the analyte or variations in the environmental conditions.

Here, the radius of the annular photonic crystal structure as shown in Fig. 1 can affect the performance of a salinity sensor based on this design. The radius determines the dimensions and periodicity of the photonic crystal, which in turn influences the propagation of light and the sensitivity of the sensor to changes in salinity. The radius of the annular photonic crystal affects the bandgap properties of the structure. The size of the bandgap is determined by the periodicity of the crystal lattice, which is influenced by the radius. A larger radius typically leads to a wider bandgap, while a smaller radius results in a narrower bandgap. The bandgap properties play a crucial role in the sensor's performance as a salinity sensor. Changes in the refractive index of the surrounding medium (in this case, seawater with varying salinity) can cause shifts in the bandgap wavelength^35,36^. By monitoring the shift in the bandgap wavelength, the sensor can infer changes in the salinity of the surrounding environment. The sensitivity of the sensor to changes in salinity can be influenced by the radius of the annular photonic crystal. A smaller radius, leading to a narrower bandgap, may result in higher sensitivity due to a larger shift in the bandgap wavelength for a given change in salinity. Conversely, a larger radius with a wider bandgap may result in lower sensitivity. Thus, the choice of radius in the design of the annular photonic crystal structure for a salinity sensor should be carefully considered to optimize the sensor's performance and sensitivity to changes in salinity^37,38^.

**Figure 2:**
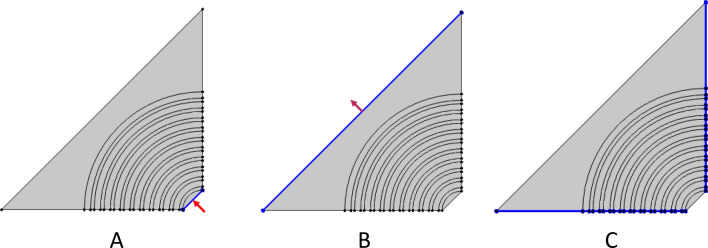
Schematic structure for 1D annular PCs with boundary conditions, since (**A**) is port 1, which is considered the direction of the incident photons, (**B**) is port 2, which is considered the transmitted electromagnetic waves, and (**C**) is the scattering boundary conditions.

**Figure 3:**
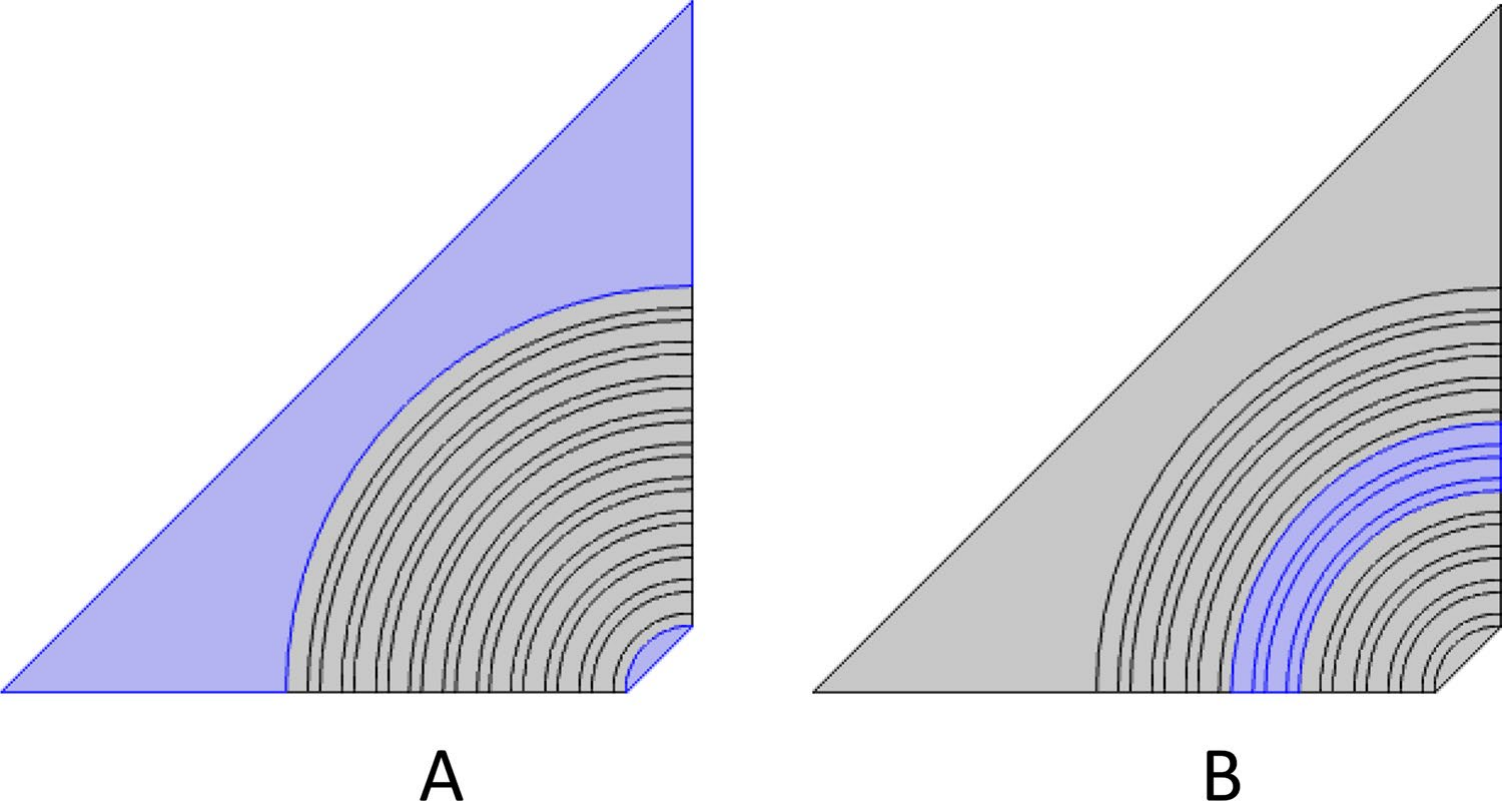
Schematic structure for 1D annular PCs with boundary conditions, since (**A**) is the perfect matching layer and (**B**) is the center layer of the structure which filled with salinity water.

Hence, we divided the last structure into four quartiles, as shown in Fig. 2, to save time during simulation. Also, for more precise results, the mesh size should be 10 times smaller than the smallest incidence wavelength. Therefore, we adjust the boundary condition for the structure as shown in Fig. 2. Wherein Fig. 2A represents the incident electromagnetic waves, Fig. 2B represents the transmitted electromagnetic waves, and Fig. 2C represents the scattering boundary conditions. Also, Fig. 3A refers to the perfect matching layers from air for the incident and transmitted waves, and Fig. 3B represents the defect layer from saline water.

**Figure 4:**
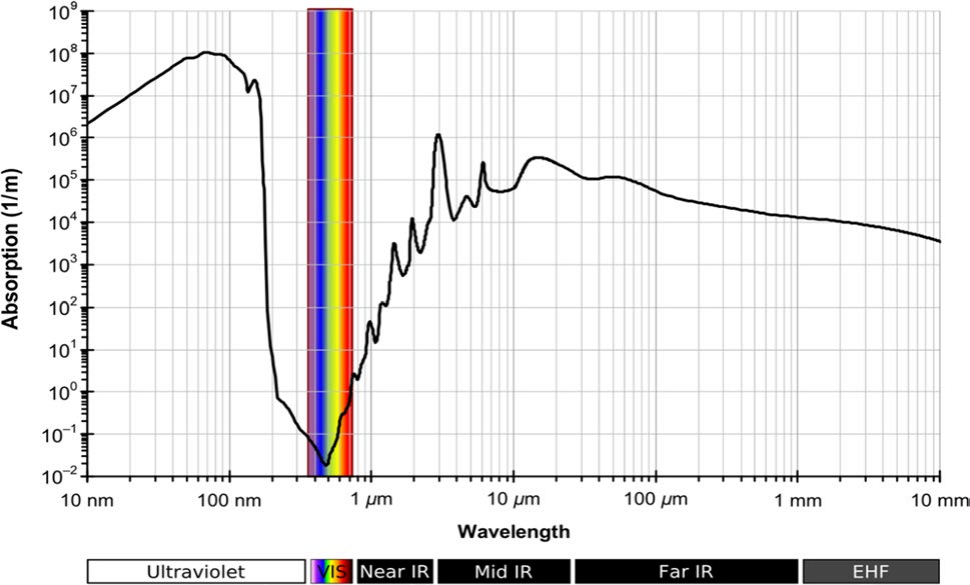
Spectrum of water absorption according to incident wavelength^43^.

Hence, we examine the optical characteristics of water in this part. It is well known that water's refractive index varies between 1.15 and 1.5 RIU and depends on the incident wavelength. As a result, fresh water has a refractive index of around 1.33 RIU at visible light^39^. Moreover, water's extension coefficient varies according to wavelength. Also, the water's absorption spectrum, given in Fig. 4, supports the idea that water is transparent to the ultraviolet and visible light spectrums due to its low absorption at these wavelengths. However, in the mid- and far-IR spectrums, the water is also substantially absorbed. Salinity (S), which is defined as the quantity of salt in grams dissolved in one kilogram of saltwater and represented in parts per thousand (PPT)^40^, indicates the amount of salt in seawater. The open ocean's salinities have been observed to be between 34 and 37 PPT, which may also be expressed as 34 to 37 practical salinity units (PSU). Wherein, seawater with S equal to 35 contains approximately 35 g of salt and 965 g of water, or 35 ppt (35 PSU). Hence, the water could be used for irrigation and human consumption at S ≤ 0.5 (PPT)^41,42^. As a result, the amount of dissolved salt in water causes a variation in the refractive index from 1.3326 to 1.3505 RIU. Hence, we evaluate the capability of our structure to describe the different refractive indices of saline water as an estimate of the salinity level.

It is important to note that the relationship between salinity and refractive index can be influenced by other factors such as temperature, pressure, and dissolved substances. Thus, as shown in Eq. (1), the refractive index of water is dependent on the following parameters: salinity S (%), seawater temperature (T), and probing wavelength ($\lambda$) in nm^44,45^.

1 $$n\left(S,T,\lambda \right)=1.314+\left(1.779\times {10}^{-4}-1.05\times {10}^{-6}T+1.6\times {10}^{-8}{T}^{2}\right)S-2.02\times {10}^{-6}{T}^{2}+\left(\frac{15.868+0.01155S-0.00423T}{\lambda }\right)-\left(\frac{4382}{{\lambda }^{2}}\right)+\left(\frac{1.1455\times {10}^{-6}}{{\lambda }^{3}}\right)$$

where, S, T, $\lambda$ and n denote the salinity (%), temperature (°C), probing wavelength in nm, and refractive index of the saltwater expressed in refractive index units (RIU), respectively.

According to the device structure for a 1D-anuular PC structure shown in Fig. 1, we have many parameters that have an impact on the performance of the device, such as the types of materials used ($${n}_{1}, { n}_{2})$$, the design dimensions (e.g., the cylindrical radius of the center of the device (r), the thickness of each layer ($${d}_{1}, { d}_{2})$$, the number of periods (N)), and the thickness of the defect layer $$({d}_{w})$$. Therefore, in the following part of results and discussions, we study the effect of these last parameters on the sensor performance.

## Results and discussions

**Figure 5:**
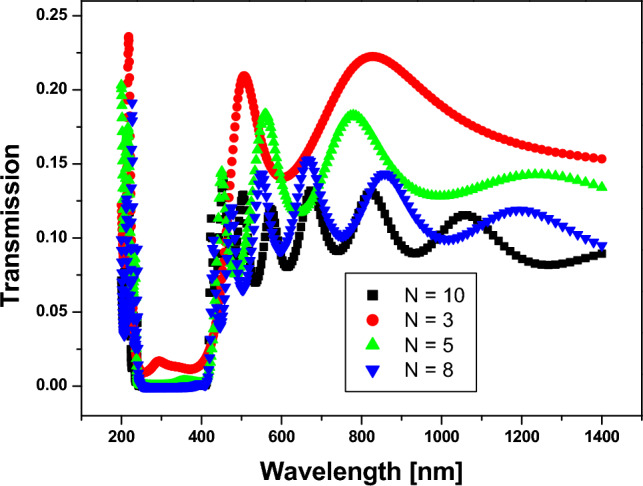
Transmittance spectrum of 1D annular PCs that consist of $Si$ and $Si{o}_{2}$ with the thicknesses of the materials represented by 25 nm and 50 nm, respectively. At different numbers of periods (N).

**Figure 6:**
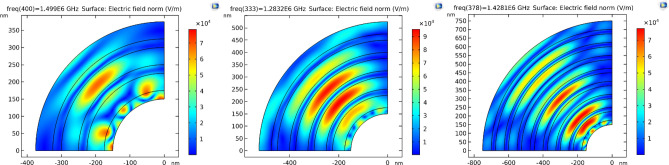
The electric field distribution through the considered structure at different number of periods as shown where (**A**) N equals 3, (**B**) N equals 5, and (**C**) N equals 8.

In this part, we show the results of our design for a 1D-anuular PCs structure. First, we optimize the structure to yield a PBG, as shown in Fig. 5. In Fig. 5, we construct a one-dimensional annular PCs structure in the form ${(AB)}^{N}$, where A is silicon ($Si$) with a refractive index $\left({n}_{1}\right)=3.3$^46^, and B is silicon dioxide ($Si{o}_{2}$) with a refractive index $\left({n}_{2}\right)=1.46$^47^. The thicknesses are donated by ${d}_{1}=25 nm$, and ${d}_{2}=50 nm$ respectively. Thus, we discovered that PBG appears at a number of periods equal to three; thus, increasing the number of periods increases the sharpness of the PBG edges, as shown, as does increasing the number of resonance peaks. Moreover, we illustrate in Fig. 6 the electric field distribution through the considered structure at different numbers of periods. In Fig. 6, we found that the electric field is localized to the silicon dioxide layer as it is the low refractive index layer; therefore, by replacing any layer with saline water, the electric field is localized to it as the refractive index of water is smaller than the refractive index of silicon dioxide.

**Figure 7:**
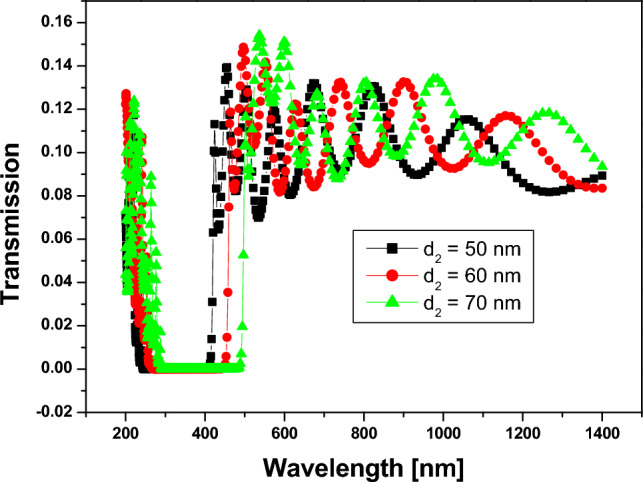
Transmittance spectrum of 1D annular -PCs that consist of $Si$ and $Si{o}_{2}$ with number of periods equal to 10, the thickness of $Si$ is denoted by 25 nm, with different thicknesses of $Si{o}_{2}$ layers.

In Fig. 7, we investigate the effect of silicon dioxide thickness on the position and width of the PBG. Hence, by increasing the thickness of $Si{o}_{2}$, the position of the PBG is shifted towards the longer wavelengths, as shown, and the width also increases by increasing the thickness of $Si{o}_{2}$, as shown in Table 1. Therefore, we chose to use the larger thickness to get a wider PBG.

**Table 1:** The width and position of PBG with varying the thickness of $Si{o}_{2}$ layer.

${d}_{1}(Si)$ nm	${d}_{2}(Si{o}_{2})$ nm	Photonic band gap		
		From $(nm)$	To $(nm)$	Width $(nm)$
25	$50$	232	412	180
25	60	255	451	196
25	70	286	490	204

**Figure 8:**
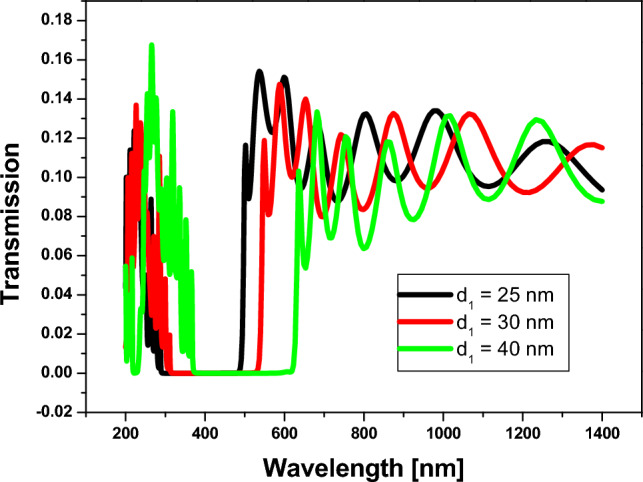
Transmittance spectrum of 1D annular PCs that consist of $Si$ and $Si{o}_{2}$ with number of periods equaling 10, the thickness of $Si{o}_{2}$ is denoted by 70 nm, with different thicknesses of $Si$ layer.

Then, we study the effect of the thickness of the silicon layer on the position and width of the PBG. Here, by increasing the thickness of $Si$, the position of the PBG is shifted towards the longer wavelengths, as shown in Fig. 8, and the width also increases by increasing the thickness of the $Si$ layer, as shown in Table 2. Thus, we chose to use the larger thickness to get a wider PBG. Therefore, we used the consistent materials of 1D annular PCs that consist of $Si$ and $Si{o}_{2}$, with the thicknesses denoted by 40 nm and 70 nm, respectively.

**Table 2:** The width and position of PBG with varying the thickness of ${\varvec{S}}{\varvec{i}}$ layer.

${d}_{1}(Si)$ nm	${d}_{2}(Si{o}_{2})$ nm	Photonic band gap		
		From $(nm)$	To $(nm)$	Width $(nm)$
25	70	286	486	200
30	70	296	540	247
40	70	364	617	253

In general, increasing the thickness of the $Si$ layer in a 1D PC structure can result in larger wavelength shifts for a given change in the surrounding medium's refractive index. Similarly, increasing the $Si{O}_{2}$ layer thickness can also lead to larger wavelength shifts. However, it's important to note that the relationship between the thicknesses and the wavelength shift is not linear and can be influenced by other factors such as the number of periods, the design of the defect layer, and the specific operating conditions. Hence, by varying the thickness of $Si{O}_{2}$ at ${d}_{Si}=25 nm$, as in Fig. 7, We have the maximum and minimum wavelength shifts for the lift PBG edge at 23 nm and 54 nm, respectively. Also, by varying the thickness of $Si$ at ${d}_{Si{O}_{2}}=70 nm$, as in Fig. 8, we have the maximum and minimum wavelength shifts of $10 nm$ and $78 nm$, respectively.

**Figure 9:**
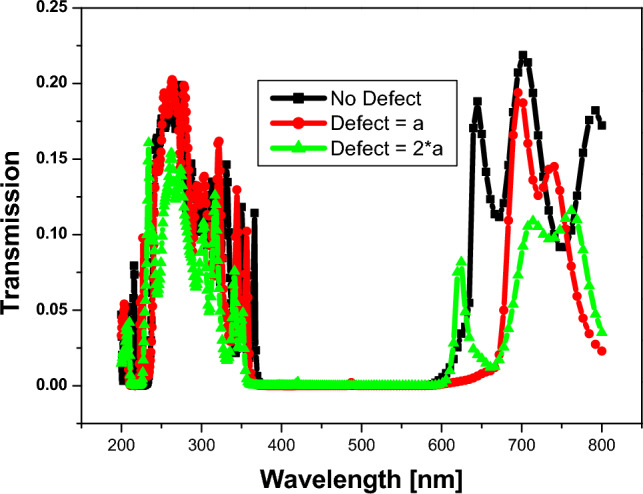
Transmittance spectrum of 9 periods of 1D annular PCs that consist of $Si$ and $Si{o}_{2}$ with the thicknesses of the materials represented by 40 nm and 70 nm, respectively. The defect layer has a variable thickness and a core defect layer of saline water with a refractive index equal to 1.3326.

Here, we used the optimum structure from 1D annular PCs that consists of $Si$ and $Si{o}_{2}$ with the thicknesses of the materials denoted by 40 nm and 70 nm, respectively, with the number of periods equaling 9, and we inserted a central defect layer from saline water with different thicknesses as shown in Fig. 9. In Fig. 9, the PBG for the structure without any defects is extended from 370 to 615 nm, with a width of 245 nm. By inserting a defect layer from saline water with a refractive index equal to 1.3326 and a thickness equal to the lattice parameter of the structure (110 nm), the PBG is extended from 362 to 674 nm with a width equal to 312 nm, and by increasing the thickness of the defect layer to 220 nm, the PBG is extended from 350 to 665 nm, with a defect peak at 623 nm.

**Figure 10:**
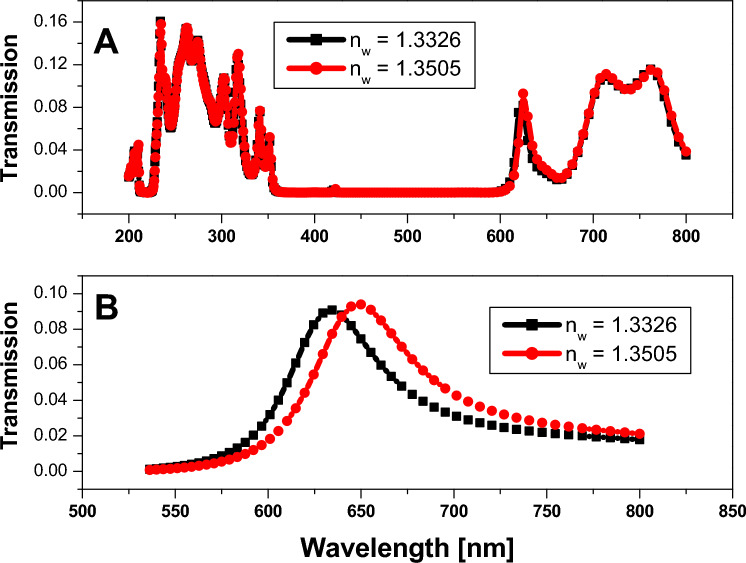
Transmittance spectrum of 9 periods of 1D annular PCs that consist of $Si$ and $Si{o}_{2}$ with the thicknesses of the materials represented by 40 nm and 70 nm, respectively. The defect layer has a variable refractive index and a core defect layer of saline water with a thickness of 220 nm.

Hence, the only case for the structure that yields a defect peak inside the photonic band gap region is that the defect layer thickness is equal to 220 nm, as shown in Fig. 9. Therefore, we choose the last structure with a defect layer equal to 220 nm, and we change the refractive index for the saline water from 1.3326 to 1.3505 to examine the performance of the sensor for changing the refractive index, as shown in Fig. 10. Figure 10A represents the transmission spectrum of the considered structure with a defect layer equal to 220 nm, wherein we have the only difference owing to the change in refractive index at the defect peak as shown. Therefore, we zoom in on this range of the defect peak, as shown in Fig. 10B, to differentiate between the two refractive indices and to calculate and determine the performance of the sensor.

The values of several factors, such as the sensitivity (S), the figure of merits (FOM), and the quality factor (Q), impact the effectiveness and performance of any sensor type. The following formulas are frequently used to determine these parameters^48,49^.

2 $$S=\frac{\Delta \lambda }{\Delta n}$$

3 $$Q=\frac{{\lambda }_{r}}{FWHM}$$

4 $$FOM=\frac{S}{FWHM}$$

where, $\Delta n,$ $\Delta \lambda$, and ${\lambda }_{r}$ are the change in refractive index, the differences in wavelengths, and the central wavelength, respectively. The full wave at half maximum is known as FWHM.

$$S=\frac{[648-634]}{[1.3505-1.3326]}=782 (nm/RIU)$$

$$Q=\frac{634}{[671-609]}=10.22$$

$$FOM=\frac{S}{[671-609]}=12.6 {RIU}^{-1}$$

**Figure 11:**
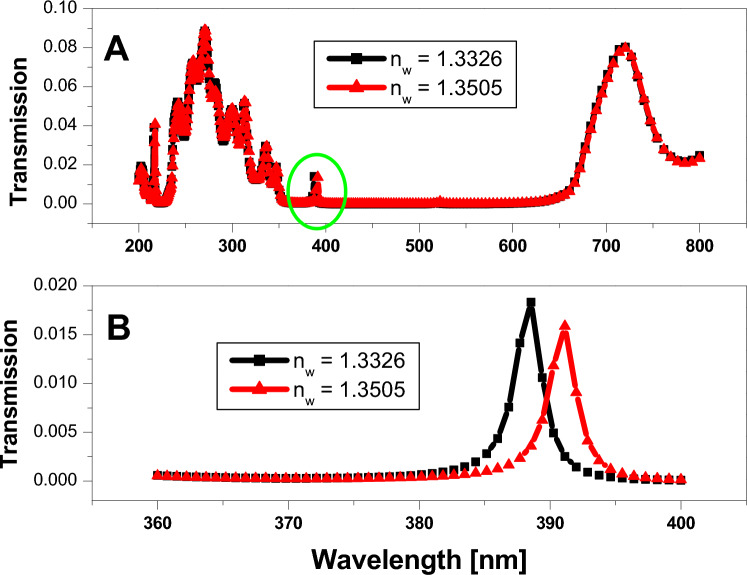
Transmittance spectrum of 9 periods of 1D annular PCs that consist of $Si$ and $Si{o}_{2}$ with the thicknesses of the materials represented by 40 nm and 70 nm, respectively. The defect layer has a variable refractive index and a core defect layer of saline water with a thickness of 330 nm.

Figure 11A represents the sensor for the defect layer thickness, which equals three folds from the lattice parameters (330 nm), and the only difference between the two different refractive indices is the small defect peaks that appear in the PBG region (the green circle). Therefore, we zoom in on these peaks, as shown in Fig. 11B, to examine the performance of the sensor. Wherein the sensitivity equals $139.6 (nm/RIU)$, the quality factor equals 170, and the figure of merit equals $60.7 {RIU}^{-1}$. As the following calculations show.

$$Sensitivity (S)=\frac{\left[391-388.5\right]}{\left[1.3505-1.3326\right]}=139.6 (nm/RIU)$$

$$Q=\frac{391}{[392.2-389.9]}=170$$

$$FOM=\frac{S}{[392.2-389.9]}=60.7 {RIU}^{-1}$$

**Figure 12:**
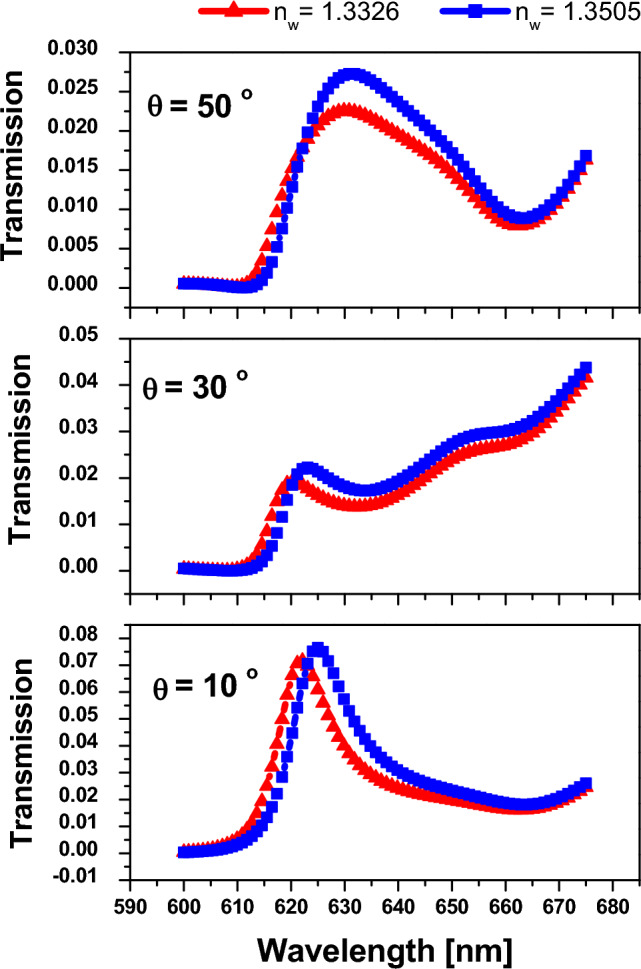
Transmittance spectrum of 1D annular PCs as in Fig. 10 at different incident angles.

Finally, we investigate the impact of incident angle on the sensor performance with the thickness of the defect layer equal to 220 nm to differentiate between the different refractive indices, as shown in Fig. 12. In Fig. 12, we found that by increasing the incident angle, the sensor's performance decreased. Therefore, the best performance is achieved at normal incidence for a saline water layer thickness of 220 nm. Thereby, we summarize the previous results of Figs. 10 and 11 as shown in Table 3 to illustrate the effect of defect thickness at normal incidence.

**Table 3:** Comparison between the performance of the proposed design as shown in Figs. 10 and 11.

Structure	Defect thickness [nm]	Period	S $(\frac{nm}{RIU})$	Q	FOM ${(RIU}^{-1})$
1D-Annular PCs	330	9	139	170	60.4
	220	9	782	10.22	12.6

Hence, from Table 3, we have found that the sensitivity strongly depends on the thickness of the defect layer in saline water. Wherein the optimum performance of the proposed salinity sensor is achieved when the defect layer thickness is equal to 220 nm owing to our optimization procedure as in Refs.^50,51^.

To illustrate the perfection of the proposed work, we finally compared the numerical values of S, Q, and FoM of our design with the same type of study work published in numerous prestigious journals. Table 4 presents the comparison information. In Table 4, we compare the different types of salinity sensors. First, we insert the results of different structures of the planner, and we find that the maximum sensitivity is achieved at the Saw Tooth structure, which equals 612.3 nm/RIU. Then, we compare our work with the previous work on the same topic of 1D-annular PCs, and we find that our structure has the optimum performance as the sensitivity equals 782 (nm/RIU). Hence, the statistics in Table 4 make it abundantly evident that the sensitivity of the salinity sensor constructed of 1D-annular PCs is greatly improved when compared to the sensitivity of the salinity sensor made of planar structures with different surface morphologies. In addition, compared to the salinity sensor formed of planar photonic structures, the quality factor and figure of merit values of the suggested structure have increased. This contrast supports our claim that a salinity sensor formed of annular PCs might be preferable to one made of planar structures. Additionally, there is a chance to investigate this work further to create alternative annular PC-based sensing systems with the intention of researching different fluids to be used as biosensors.

**Table 4:** Compares the proposed design with past works by other photonic researchers, highlighting the suggested design's sensitivity, quality factor, and FoM values.

	Structure	Design	Reference	Year	S $(\frac{nm}{RIU})$	Q	FOM ${(RIU}^{-1})$
One-dimensional PCs	Ordinary		^52^	2022	559.7	2882	1998
	Texturing		^52^	2022	569	236	170
	Saw tooth		^52^	2022	612.3	272.4	199
	Annular		^53^	2017	17	$3\times {10}^{4}$	$2.2\times {10}^{2}$
			^54^	2020	10	$3\times {10}^{2}$	15.1
			^55^	2021	640	$1.5\times {10}^{5}$	$2.6\times {10}^{4}$
			^56^	2023	472.79	$8\times {10}^{3}$	$5.2\times {10}^{3}$
			Current work		782	10.22	12.6

## Conclusion

In this paper, we succeeded in building up a 1D annular PCs structure in the form ${(AB)}^{N}$, where A and B are $Si$ and $Si{o}_{2}$, with the materials' thicknesses indicated by 40 nm and 70 nm, respectively, $N=9$, by including a layer of central defect of saline water $({d}_{w}=220 nm)$. We used COMSOL Multiphysics as a simulation tool, which depends on the FEM. Therefore, we get the optimum sensor performance as follows: the sensitivity (S) equals $782 (nm/RIU)$, the quality factor (Q) equals 10.22, and the figure of merit (FOM) equals $12.6 {RIU}^{-1}$. We compare our structure of 1D annular PCs with different structures of 1D-PCs such as planners, texturing, and saw tooth structures from recently published papers, as shown in Table 4. In addition, we compare our design with different structures of the same type (1D annular PCs). Hence, the findings presented in this paper contribute to the advancement of photonic crystal-based sensing technologies and pave the way for future developments in the field of salinity sensing, as annular 1D photonic crystals hold great promise for salinity sensing applications.

## Data Availability

The datasets used and/or analyzed during the current study are available from the corresponding author on reasonable request.
